# Bridging 3D Slicer and ROS2 for Image-Guided Robotic Interventions

**DOI:** 10.3390/s22145336

**Published:** 2022-07-17

**Authors:** Laura Connolly, Anton Deguet, Simon Leonard, Junichi Tokuda, Tamas Ungi, Axel Krieger, Peter Kazanzides, Parvin Mousavi, Gabor Fichtinger, Russell H. Taylor

**Affiliations:** 1 Whiting School of Engineering, Johns Hopkins University, Baltimore, MD 21218, USA; anton.deguet@jhu.edu (A.D.); sleonard@jhu.edu (S.L.); axel@jhu.edu (A.K.); pkaz@jhu.edu (P.K.); rht@jhu.edu (R.H.T.); 2 School of Computing, Queen’s University, Kingston, ON K7L 3N6, Canada; ungi@queensu.ca (T.U.); mousavi@queensu.ca (P.M.); fichting@queensu.ca (G.F.); 3 Brigham and Women’s Hospital, Boston, MA 02115, USA; tokuda@bwh.harvard.edu

**Keywords:** ROS, 3D Slicer, image-guided therapy, robotics, software, prototyping

## Abstract

Developing image-guided robotic systems requires access to flexible, open-source software. For image guidance, the open-source medical imaging platform 3D Slicer is one of the most adopted tools that can be used for research and prototyping. Similarly, for robotics, the open-source middleware suite robot operating system (ROS) is the standard development framework. In the past, there have been several “ad hoc” attempts made to bridge both tools; however, they are all reliant on middleware and custom interfaces. Additionally, none of these attempts have been successful in bridging access to the full suite of tools provided by ROS or 3D Slicer. Therefore, in this paper, we present the SlicerROS2 module, which was designed for the direct use of ROS2 packages and libraries within 3D Slicer. The module was developed to enable real-time visualization of robots, accommodate different robot configurations, and facilitate data transfer in both directions (between ROS and Slicer). We demonstrate the system on multiple robots with different configurations, evaluate the system performance and discuss an image-guided robotic intervention that can be prototyped with this module. This module can serve as a starting point for clinical system development that reduces the need for custom interfaces and time-intensive platform setup.

## 1. Introduction

Over the last three decades, medical robots have been used to augment human capabilities in a clinical environment in several ways, such as offering high dexterity and precision in hard-to-reach places [1], correcting human inaccuracy like hand tremors [2], or reducing the invasiveness of surgical procedures [3]. Currently, there are several approved medical robots used in regular practice, including the da Vinci robot [4] for laparoscopic surgery, the Mako robot [5] for orthopedic surgery, and the CyberKnife robot for radiosurgery [6]. Alongside these advancements in medical robotics, the application of surgical navigation and image-guided therapy (IGT) has also experienced rapid growth and clinical deployment [7]. In fact, the use of surgical navigation systems to execute pre-defined plans based on imaging is now commonplace for a variety of surgical procedures [8]. An example of this technology is the LumpNav system for breast-conserving surgery (BCS) [9] or the iASSIST for total knee replacement (TKR) [10]. Naturally, image-guided robotics is an emerging area of research for procedures that require both active imaging and accurate tool placement, such as laparoscopic radical prostatectomy [11] or needle ablation [12]. Discovering new clinical applications for image-guided robotics is, therefore, an active area of research.

Research on these new applications requires access to hardware and compatible software tools. Acquiring these from commercial distributors can be difficult because of software licensing and the high up-front costs associated with new systems. Additionally, the proprietary nature of some of these products prevents researchers from gaining access to the entire software and hardware interface. As a result, there have been efforts made to offer open-source software development tools in both the medical and broader robotics industry [13]. Within the robotics community, one of the most commonly used tools for this is the robot operating system (ROS) which is an open-source control solution for robotics [14]. One of the major benefits of ROS is that it has a number of already implemented features that are highly useful for medical robotics, including motion planning, kinematics, and simulation [11]. Moreover, ROS interfaces are provided for research systems and devices that closely resemble a clinical system like the da Vinci Research Kit (dVRK) [15] and the KUKA lightweight robot (LWR) [16]. The recent release of ROS2 introduced Windows and Mac support (which was not previously available) and has made ROS even more useful by accommodating programmers across operating systems.

In congruence with the medical robotics research community, there has also been a push to develop open-source platforms and tools to enable IGT research [17]. One of the most popular and well-maintained tools within this domain is the open-source medical imaging platform 3D Slicer [18]. The 3D Slicer platform uses a community development approach to support a variety of toolkits and extensions that can be used to prototype new IGT applications. There are several extensions within 3D Slicer that support clinical computation, such as segmentation [19], tracking [20], volume reconstruction [21], and now artificial intelligence (AI) development [22].

**Figure 1:**
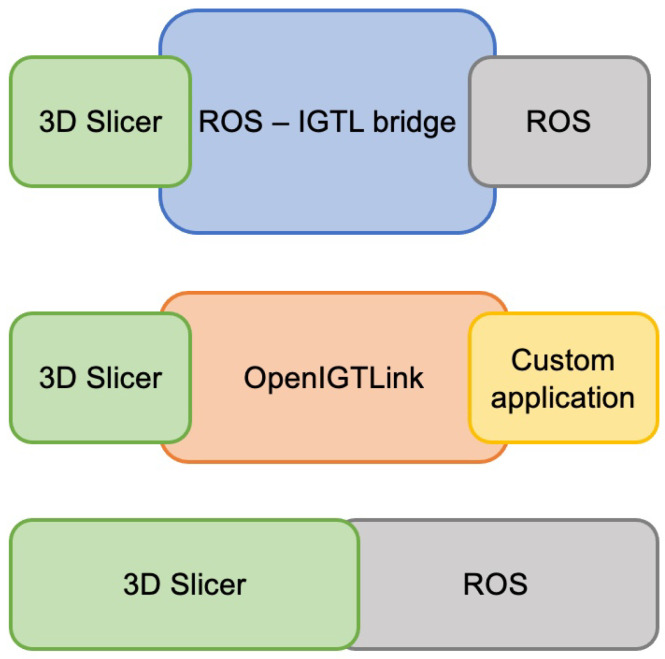
Comparison of existing methods to bridge ROS and 3D Slicer. (**Top**) ROS-IGTL bridge configuration. (**Middle**) OmniIGT configuration. (**Bottom**) Proposed SlicerROS2 configuration.

At present, ROS requires custom development to accommodate the imaging, localization, and navigation that is necessary for surgical applications. As well, 3D Slicer does not have a simple interface for robotics. There have been a few approaches taken to bridge the two tools (ROS and 3D Slicer) using a communication protocol called OpenIGTLink [23]. Namely, the ROS-IGTL bridge [11] uses OpenIGTLink to stream data between 3D Slicer and ROS (Figure 1, top). This bridge can be used on any robot with a supported ROS interface but still requires two network hops, customization on the 3D Slicer side, and is limited in the types of messages that can currently be sent. Additionally, custom 3D Slicer and robotics interfaces with OpenIGTLink have been developed—such as the OmniIGT system [24] and the LightweightRobotIGT system [16] (Figure 1, middle).

**Figure 2:**
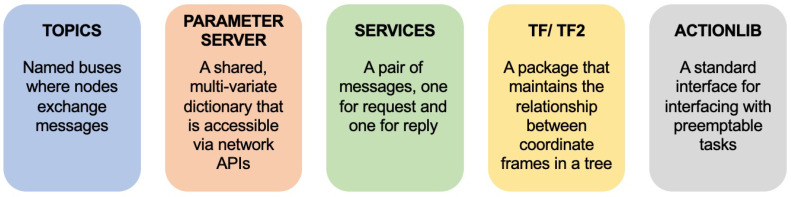
ROS resources that are commonly used for the development of medical robotic systems. Definitions taken from ROS wiki pages [14].

Although these approaches are useful for sharing data elements like models, transforms, etc., they do not share full context information such as the kinematics of the robot or the 3D Slicer scene graph. For example, the OmniIGT module facilitates visualization of a robot in 3D Slicer and simple control (incremental motion in each direction) but it does not compute the kinematics of the robot or offer translation of the 3D Slicer scene graph to the robot. The reliance of each of these methods on middleware also makes it difficult to provide access to the full range of ROS libraries and resources, illustrated in Figure 2.

Each of these resources would need to bridged to 3D Slicer differently. For example, the ROS-IGTL bridge can stream topics to OpenIGTLink currently but not services. Bridging the entirety of ROS to another ecosystem has been done before with the Orocos Real-Time Toolkit (RTT) for advanced robotic control [25], but would require substantial development efforts and resources. Moreover, the use of any middleware also adds latency due to computing cost, serialization, and deserialization, and handling queues of messages. Even if this added latency doesn’t affect the performance or usability of a system, eliminating the middleware can potentially reduce the probability of system failure and improve the patient safety.

Therefore, in this paper, we demonstrate an initial offering of the SlicerROS2 module, which was developed for open-source prototyping of ROS2 compatible systems in 3D Slicer. We treat 3D Slicer as a node within ROS and therefore create a system that can leverage the benefits of both infrastructures in a single network hop (Figure 1, bottom). The module was designed to relay contextual information between the imaging and robotics domains while considering ease of use and generalizability. We show that the new application can offer (1) low latency visualization of robots in the Slicer 3D viewer, (2) generalizability to robots with both serial and parallel linkage, and finally, (3) translate the transformation hierarchy that is employed by 3D Slicer to that of ROS. We also demonstrate a proof of concept application that requires spatial registration of anatomy to a robot and temporal synchronization to show how this system may facilitate future research.

## 2. Methods

**Figure 3:**
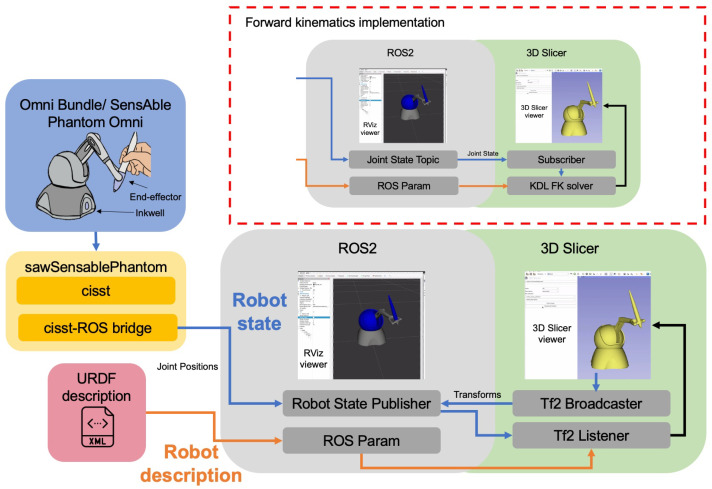
Overview diagram of the SlicerROS2 module. Green boxes indicate 3D Slicer features, while gray indicates ROS2 features. Blue arrows indicate connections specific to robot state, while orange indicates connections specific to robot description. The URDF description is loaded into ROS param to update the robot description in 3D Slicer. At the same time, the robot state publisher updates the Tf2 tree in ROS for 3D Slicer to listen and broadcast to. The upper right shows the same configuration using the other robot state alternative, which is computing the FK of the device directly in 3D Slicer.

The SlicerROS2 module is built to enable direct access to ROS2 nodes within 3D Slicer. ROS2 is chosen over ROS1 because, by 2025, the ROS development community should be fully migrated from ROS1 to ROS2 [26]. We focus on data transfer between the Slicer Medical Reality Modelling Language (MRML) [27] scene to the ROS transformation hierarchy (Tf2, formerly known as Tf) [28]. An overview of this approach is demonstrated in Figure 3.

**Figure 4:**
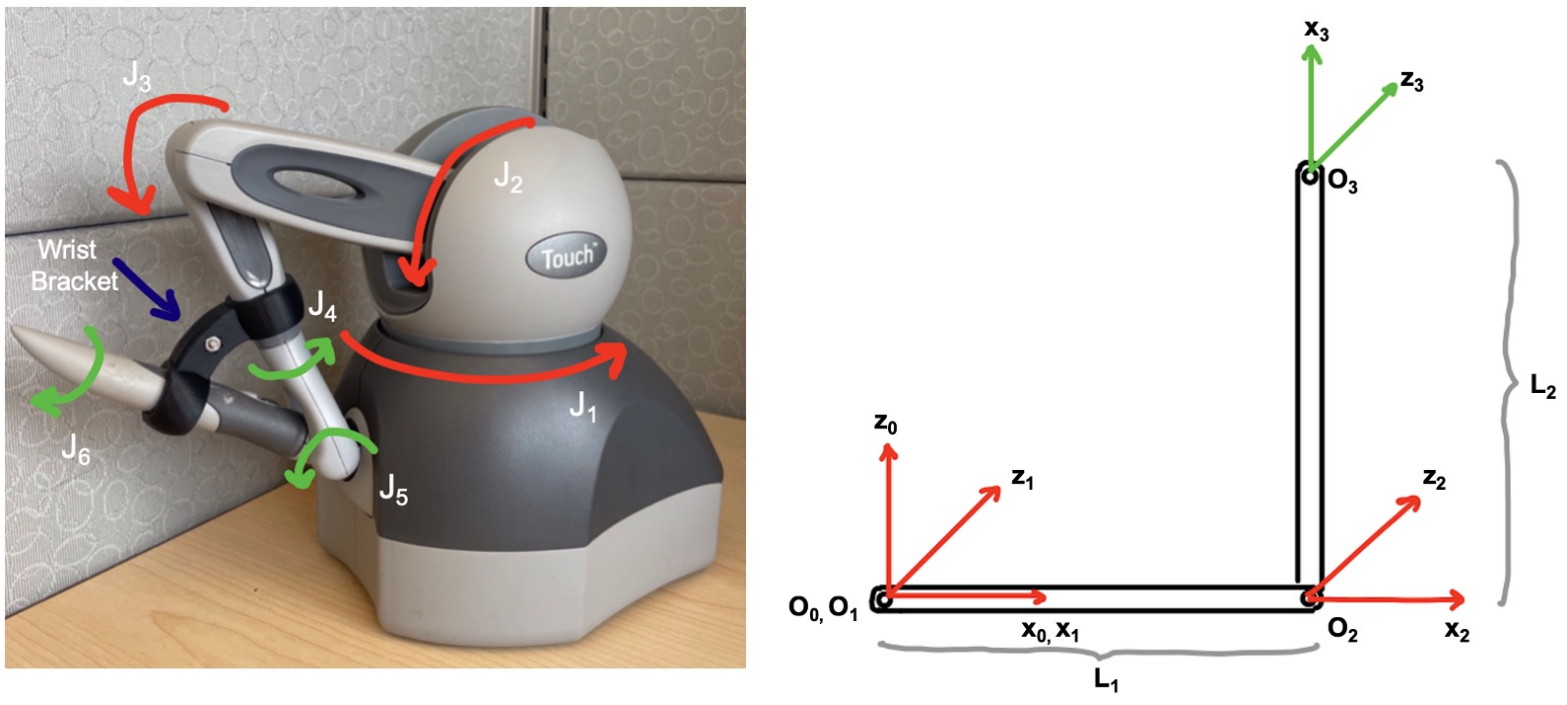
Omni bundle robot used for prototyping the SlicerROS2 module. Previously known as the SensAble Phantom Omni or Geomagic/3DS Touch. Joints labeled in red are actuated, while joints labeled in green are not. (**Left**) Labeled device. (**Right**) Device coordinate frames such that L1 and L2 are the link lengths.

The following sections explain this implementation in more detail. For simplicity, we describe the use of the SlicerROS2 module on the Omni Bundle robot (previously known as the SensAble Phantom Omni), which is used for prototyping (Figure 4).

### 2.1. Comparison to Previous Implementations

As mentioned in the introduction, there have been previous implementations of robotics research platforms in 3D Slicer, one of which is the OmniIGT platform that was developed for the same device [24]. In this implementation, the same software package, sawSensablePhantom, is used to interface with the device. To get this information from the device to 3D Slicer, sawOpenIGTLink (www.github.com/jhu-saw/sawOpenIGTLink—accessed on 20 June 2022), which can be used with any collaborative robotics toolkit (CRTK) compatible interface, is used to stream information over OpenIGTLink (www.openigtlink.org—accessed on 20 June 2022) [23]. The OpenIGTLink interface (OpenIGTLinkIF) allows us to read and write this information to and from 3D Slicer.

**Figure 5:**
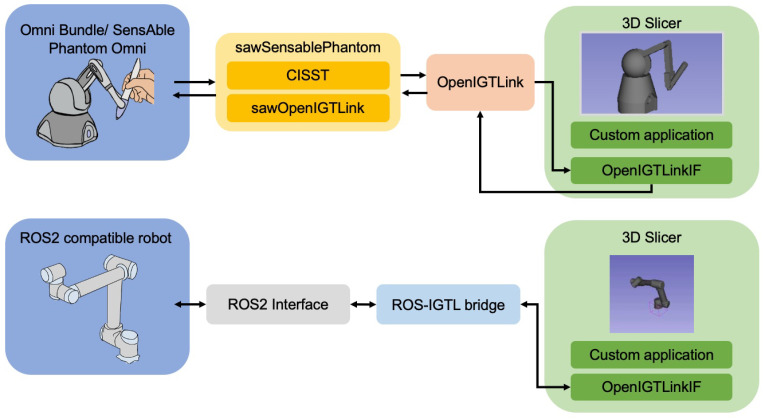
Detailed overview of previous implementations. (**Top**) OmniIGT platform [24] demonstrates the use of a custom application. (**Bottom**) ROS-IGTL bridge demonstrates the use of the more general bridge infrastructure [11].

Similarly, the ROS-IGTL bridge has been implemented for message transfer between ROS and OpenIGTLink. Instead of requiring a custom interface to read joint values from the device, the ROS-IGTL bridge is set up to interface directly with ROS-compatible devices. Messages can be sent from ROS to OpenIGTLink with the bridge and vice versa. Figure 5 describes both of these approaches in more detail.

### 2.2. Requirements

The SlicerROS2 module is designed to facilitate the prototyping of image-guided robotic systems and therefore has a set of functional requirements to meet the needs of clinical research. These requirements demonstrate real-time visualization of robots in 3D Slicer and enable data transfer in both directions between Tf2 and the Slicer MRML scene. Additionally, the platform should be able to accommodate any robot supported in ROS, to limit the need for custom applications or interfaces, manual intervention, or any device specific implementations.

A loadable module programmed in C++ was developed to meet these requirements. The module uses the colcon tool to build packages that are based on the ament toolset and standard for ROS2 development [26]. The process is similar to that of CMake and is necessary for setting up the environment for a package and invoking the build with ROS2. In this configuration, we essentially treat the loadable 3D Slicer module as a ROS node.

### 2.3. Dependencies

The SlicerROS2 module uses rclcpp (www.github.com/ros2/rclcpp—accessed on 20 June 2022) which is the C++ API for ROS2. The use of rclcpp allows us to create a ROS node for the module on startup, which can be used to create subscriptions and publish messages. Beyond rclcpp, the SlicerROS2 module also uses the universal robot description format (URDF) package (www.github.com/ros/urdf—accessed on 20 June 2022), the orocos kinematics and dynamics library (KDL) (www.github.com/orocos/orocos_kinematics_dynamics—accessed on 20 June 2022), and the Tf2 package. The other dependencies are standard for loadable modules (C++ plugins built against Slicer), one of which requires that the user has Slicer built from source on their computer. More detailed instructions for this process are available on the project GitHub page (www.github.com/rosmed/slicer_ros2_module—accessed on 20 June 2022).

### 2.4. Robot Description

The connections that are relevant to the robot state are highlighted in orange in Figure 3. To ensure generalizability, one of the main considerations for the module is how robots are described and loaded into 3D Slicer. The URDF format is the de-facto standard to describe the geometry of robots [29]. URDF is an xml file format that is used to specify the kinematics and dynamic properties of a robot. These descriptions can be parsed directly using the URDF library or they can be loaded from the ROS parameter server, which is designated to storing global variables and parameters. In some cases, the URDF file needs to be processed to expend some macro commands (xacro in ROS terms). ROS param can store the result of this process so users, including the SlicerROS2 module, can load the resulting URDF without having to deal with said xacros. Upon startup of the module, the user can select whether they want to load the visual from the URDF file directly or from the parameter server.

### 2.5. Robot State

The connections that are relevant to the robot state are highlighted in blue in Figure 3. The module is set up to update the robot state in two ways: either using the built-in ROS transformation package (Tf2) or calculating the forward kinematics (FK) based on the joint values of the robot. The Tf2 implementation is preferred because it supports robots with serial and parallel linkage, whereas the current FK implementation only supports robots with serial links. The latter is included to support a use-case where a user might not want to use the robot state publisher (standard ROS node), which computes the position and orientation of each link for Tf2. For the Omni Bundle robot, the sawSensablePhantom libraries (part of the cisst-SAW libraries developed at Johns Hopkins [30]) can be used to interface directly with the device and read joint states. The cisst-ROS bridge can then be used to publish these joint states as ROS topics [15]. Note that this bridge is only necessary for this implementation because, to our knowledge, there is no direct ROS2 interface for the Omni Bundle Robot. Furthermore, this implementation helps emphasize the importance of reducing middleware and bridging wherever possible.

To use Tf2, the joint states are then fed to the robot state publisher, which outputs the position and orientation of each coordinate frame to the Tf2 package. This is initialized with a python launch file (www.github.com/jhu-saw/ros2_sensable_omni_model—accessed on 20 June 2022). Tf2 then keeps track of each frame relative to the robot base at all times. Within the SlicerROS2 module, a Tf2 listener, is set up to read the transformations for the device’s links and update the MRML scene accordingly. A Tf2 broadcaster is also set up within the module to send transformations from the MRML scene in Slicer to ROS.

To use forward kinematics, a similar approach is taken, but the KDL library is used directly within the module and the Slicer node subscribes to the topic publishing the joint state. This implementation is demonstrated in the top right of Figure 3.

### 2.6. Synchronization

To update the 3D viewer in Slicer based on the actions and events happening on the ROS node, a Qt timer is set in 3D Slicer to spin the ROS node (check for event updates) and either query Tf2 or calculate the FK at a rate of 100 Hz. Running this check on a timer allows us to run the code in the Slicer main thread, which is beneficial because the processes running in the SlicerROS2 module are not computationally expensive. Using a ROS timer within 3D Slicer would require a separate synchronization method for all of the data in the scene.

## 3. Experiments

### 3.1. Generalizability

To evaluate the generalizability of the SlicerROS2 module, we tested it on two separate robots with different configurations. The first device is the Omni Bundle robot (used for prototyping the system) which is a serial link manipulator. The second robot was the dVRK patient side manipulator (PSM), which has parallel linkage [31].

The direct interface to both devices is provided by the cisst-SAW libraries. A launch file was then used to initialize the robot state publisher. The robot description (URDF) was loaded from the ROS2 parameter server and Tf2 was used to monitor the robot state. The robot was then moved through a variety of positions to evaluate the accuracy of the visualization in 3D Slicer by comparing it to that of RViz, the ROS graphical user interface (GUI) for visualization.

### 3.2. Latency Evaluation

**Figure 6:**
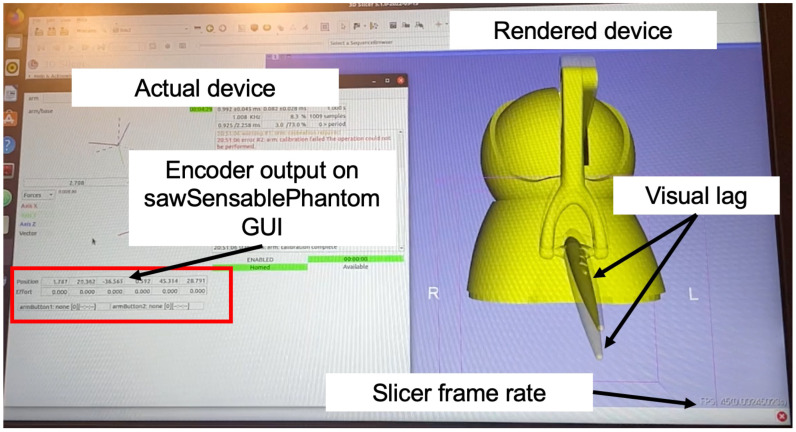
Screenshot from experiment recording: displaying the actual device encoder reading, rendered device, and an example of the visual lag used to qualify movement completion.

The latency of the visualization of the robot in 3D Slicer was also evaluated. To do so, the movement of the Omni Bundle (actual device) and the 3D visualization in Slicer (rendered device) were captured and compared with a single Apple iPhone 12 camera. Figure 6 is a screenshot of the experiment recording. The video was captured in slow motion at 2532 by 1170 pixels at a frame rate of 240 frames per second (fps).

The joint state publisher was updated at a rate of 100 Hz, while the node in 3D Slicer was spun at a rate of 100 Hz and the sawSensablePhantom code published the encoder data at a rate of 100 Hz. The end-effector (EE) of the device was then manually removed from the inkwell (shown in Figure 3—considered “home” for the device), extended, and placed back in the inkwell 15 times. The visual latency was computed by recording the time difference, in milliseconds (ms), between movement completion on the actual device versus the rendered device. The rendering completion time was detected from the recording after the experiment using the open-source, cross-platform video player mpv on Mac (www.github.com/mpv-player/mpv—accessed on 20 June 2022), where playback time can be displayed in ms. To capture, the movement of the actual device, the encoder values of the first three joints were displayed using the sawSensablePhantom GUI, shown in Figure 6. Movement completion for the actual device was considered the time when the encoder readings for the first three joints were stable (i.e., the device is homed), while movement completion for the rendered device was considered the frame/time when the rendered robot was in the expected location and the visual lag of the rendering had stopped. The frame rate of the 3D Slicer viewer was also displayed during the experiment for reference.

### 3.3. Surgical Use Case

To demonstrate the utility of this platform, we also performed an experiment using data previously recorded during surgery for integrating this robotic system with an existing surgical navigation platform called LumpNav [9]. The LumpNav system is designed to facilitate breast-conserving surgery (BCS) under EM navigation and ultrasound guidance. BCS is a procedure where the surgeon resects a tumor from the breast while attempting to preserve the surrounding healthy tissue. These procedures can be performed with wire localization, which is when a radiologist guides a needle through the tumor before the surgery begins. Currently, around 20–30% of these procedures result in positive margins, which indicates that the entire tumor was not completely removed [20]. When this happens, the patient has to undergo repeat surgery which can pose several adverse health effects.

To address this high rate of incomplete resection, the Lumpnav system introduces image guidance and navigation in BCS to provide the surgeon with visual feedback about the tumor resection margins. In this system, before the surgery starts, a radiologist uses ultrasound to create a 3D annotation of the tumor. This information is then used to construct a model in 3D Slicer that is tracked using an EM sensor which is then fixed to the end of the localization needle. Additionally, the surgeon’s tool (cautery) is also EM tracked relative to a reference EM sensor that is fixed to the patient’s chest. All of this tracking information is combined through the LumpNav module (in 3D Slicer) to provide the surgeon with real-time visualization of their tool relative to the annotated tumor margins during surgery. If the surgeon’s tool intersects with the annotated tumor model during the procedure, the tumor model turns red to indicate that the surgeon is creating a positive margin. Otherwise, the tumor model is green to indicate that the surgeon is operating outside of the annotation.

One of the current limitations of LumpNav is that the system is still heavily reliant on freehand guidance to avoid positive margins. As breast tissue is highly mobile and deformable, staying outside of these margins with only visual feedback is a challenging task. Therefore, this procedure could benefit from a virtual fixture (a cooperative control concept) that would be imposed around the annotated tumor model to prevent the surgeon from entering this area at all [32].

To show how this application could be deployed with the SlicerROS2 module, we performed an experiment with a recorded lumpectomy case where the LumpNav system was deployed. Using the SlicerROS2 module and built-in 3D Slicer tools, the robot is registered to the spatial coordinates of the surgeon’s tool and the 3D tumor model. The EE of the robot is then positioned in the same orientation as the surgeon’s tool from a recorded LumpNav case to mimic an application where the tool is cooperatively guided. We then implemented a simple code to detect the collision between the tip of the robot EE and the tumor and demonstrate what this would look like in implementation in the 3D Slicer viewer. This function uses similar logic to that of LumpNav, which measures the distance between the tip of the cautery and the surface model of the tumor. We modify this to instead consider the tip of the Omni bundle as the surgeon’s tool and use a filter that detects the collision between the surface model of the robot tip and the surface model of the tumor. This modification was made because the LumpNav implementation requires that we EM track the tip of the Omni bundle, which was not done for this use case simulation.

## 4. Results

**Figure 7:**
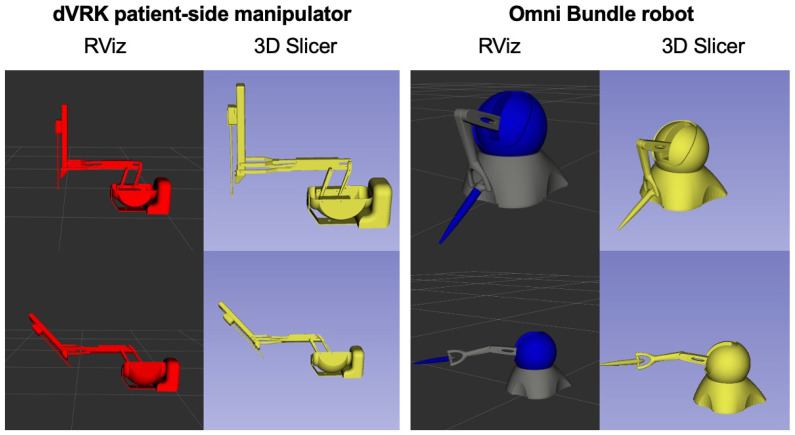
(**Left**) SlicerROS2 visualization of dVRK PSM1 in two different configurations. (**Right**) SlicerROS2 visualization of Omni Bundle robot in two different configurations. The viewer on the left is RViz, while the viewer on the right is 3D Slicer for both devices.

Figure 7 below demonstrates the visualization of the dVRK PSM and the Omni bundle robot in two different configurations.

The visualization of each device in both RViz and 3D Slicer is the same. Moreover, configurations that required movement of multiple joints were tested to ensure the module could generalize to both parallel and serial manipulator motions. In each case, the module performed as expected. The average latency of the module was also determined to be 11.2 ± 4.5 ms (*n* = 15). Considering that we use 100 Hz for all our periodic processes, this measured latency roughly corresponds to a single frame delay. During this evaluation, the frame rate of the 3D Slicer viewer was also around 42–45 fps.

**Figure 8:**
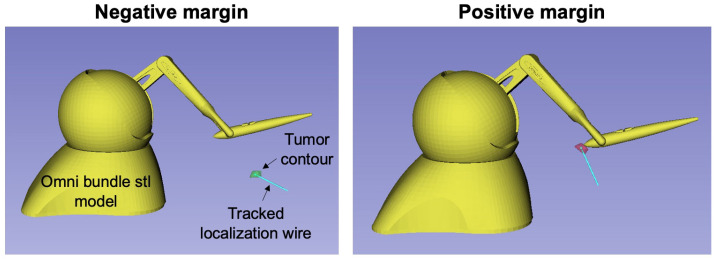
Application of cooperative robotics in previously recorded navigated lumpectomy surgery. (**Left**) Negative margin—when the robot/cooperatively guided tool is outside of the tumor. (**Right**) Positive margin—when the robot/cooperatively guided tool is inside of the tumor. Note that breast tumors are generally small [33].

The following visualization was derived from the integration of the SlicerROS2 platform with a prerecorded LumpNav case (Figure 8).

The information from LumpNav and SlicerROS2 is fused in the 3D viewer to provide a visual demonstration of the robot relative to the annotated tumor model for a negative margin (where the surgeon’s tool is outside the tumor model) and a positive margin (where the surgeon’s tool is inside the tumor model). In this scenario, the motion of the anatomy, robot, and EM navigation system are all spatially and temporally synced.

## 5. Discussion

In evaluating how the SlicerROS2 module can adapt to different robot configurations, we show that the system can be used to load two very different robots in 3D Slicer without any additional operator intervention. The user can simply specify where the description and state are coming from and the module will populate the MRML scene with the corresponding transformations automatically. In comparison to existing implementations for visualizing robots in 3D Slicer, this automatic loading is a significant improvement as other implementations require the user to attach a transform to each joint and incrementally update that transforms to get each joint to the approximate correct configuration. As well, to our knowledge, this is the first reported dynamic visualization of the dVRK PSM in 3D Slicer [34].

With respect to latency, the SlicerROS2 module had a reported latency of 11.2 ± 4.5 ms when rendering the movement of the entire robot. For performance comparison, the LightweightRobotIGT platform, which is designed to interface the KUKA robot with 3D Slicer, has a reported latency of 60 ms [16]. It’s also important to note that the latency of the LightWeightRobotIGT platform (60 ms) is only reported for rendering the EE of the device in the 3D Slicer viewer. The reported latency of SlicerROS2 (11.2 ms) is reported for rendering the entire robot in the 3D Slicer viewer. There are three computational components in SlicerROS2 that contribute to this latency: the publishing rate of robot encoder data, the publishing rate of the joint state publisher, and the rate at which the node in the 3D Slicer is spun. These were all set to 100 hz (10 ms) for this evaluation but run asynchronously. The latency results may also be influenced by the rendering frame rate in 3D Slicer (42–45 fps) and the frame rate of the camera (240 fps). Therefore, the overall latency of 11.2 ± 4.5 ms seems to be reasonable. In terms of the impact of this latency on the actual tasks, a previous study on several different human-computer interaction (HCI) tasks reported that the lowest reported accuracy that impacted performance was 16 ms [35]. This suggests that the reported latency of the SlicerROS2 module will not significantly impact the operator’s tasks in real-world applications.

**Figure 9:**
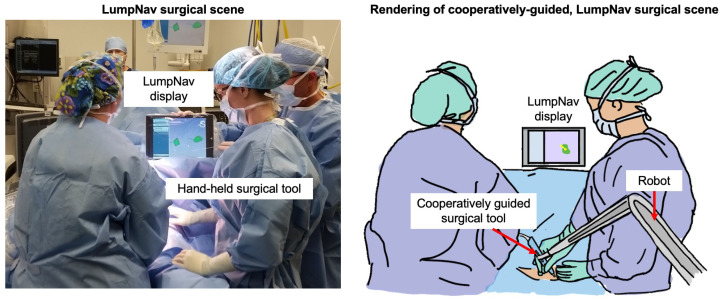
(**Left**) Surgical scene from LumpNav case with no cooperative robotic guidance. (**Right**) Rendering of the surgical scene with LumpNav and cooperative robotic guidance.

Furthermore, the application of the SlicerROS2 system in navigated BCS helps demonstrate the benefit of this prototyping platform. With the information that is already provided by the SlicerROS2 and LumpNav platform, developing a virtual fixture system for positive margin avoidance is achievable. In this application, the position of the robot can be EM tracked and registered to the position of the tumor. As the groundwork architecture for this system is completed with SlicerROS2, the development of this idea can instead be focused on the fundamental research concepts and execution. Figure 9 demonstrates what the addition of cooperative robotics to the LumpNav system might look like.

This is only one example of an application that can use the SlicerROS2 module for deployment. A more generic example is robotic needle placement in a target area based on annotated preoperative imaging. Image annotation is a common process that is done in 3D Slicer, while trajectory generation and actuation of a robot (to insert a needle) can be accomplished in ROS2. The advantage that SlicerROS2 offers for this application are that the annotated image can be registered to the live patient position and shown in the same coordinate space and 3D viewer as the robot. This information can then be used to inform the path of the needle relative to the target of interest.

Some of the limitations of the current SlicerROS2 module are that despite ROS2 being available on Linux, Windows, and Mac, this module has only been tested extensively on Linux. Using the module on other operating systems would require more testing. Moreover, the module requires that Slicer is built from the source on the computer used for deployment. The deployment of the module is also not currently set up for simple distribution in the extension manager like other 3D Slicer extensions. At this time, the SlicerROS2 module has not been used to display two robots simultaneously, which may be required by some clinical applications. The performance of the module with more than one device may be impacted by this additional complexity and, therefore, should be evaluated in the future.

## 6. Conclusions

In this paper, we present the SlicerROS2 module, designed for prototyping image-guided robotic interventions. We treat 3D Slicer like a ROS2 node to make use of the full suite of tools and packages offered by both ROS2 and 3D Slicer. We evaluate the system by showing that it is a general solution for visualizing robots, has minimal visual latency, and can be used to develop practical, image-guided robotic systems. This module can benefit the image-guided and medical robotics scientific community by serving as a starting point for clinical system development that reduces the need for custom interfaces and software platforms. Future work includes trying the SlicerROS2 module with more ROS2 compatible devices as well as potentially including more ROS libraries directly in Slicer like motion planning or simulation.

## Data Availability

The data from this study is not available.

## References

[1] Rea F., Marulli G., Bortolotti L., Feltracco P., Zuin A., Sartori F. (2006). Experience with the “da Vinci” robotic system for thymectomy in patients with myasthenia gravis: Report of 33 cases. Ann. Thorac. Surg..

[2] Taylor R., Jensen P., Whitcomb L., Barnes A., Kumar R., Stoianovici D., Gupta P., Wang Z., Dejuan E., Kavoussi L. (1999). A steady-hand robotic system for microsurgical augmentation. Int. J. Robot. Res..

[3] Troccaz J., Dagnino G., Yang G.Z. (2019). Frontiers of medical robotics: From concept to systems to clinical translation. Annu. Rev. Biomed. Eng..

[4] DiMaio S., Hanuschik M., Kreaden U. (2011). The da Vinci surgical system. Surgical Robotics.

[5] Roche M. (2021). The MAKO robotic-arm knee arthroplasty system. Arch. Orthop. Trauma Surg..

[6] Kilby W., Dooley J., Kuduvalli G., Sayeh S., Maurer C. (2010). The CyberKnife^®^ robotic radiosurgery system in 2010. Technol. Cancer Res. Treat..

[7] Cleary K., Peters T.M. (2010). Image-guided interventions: Technology review and clinical applications. Annu. Rev. Biomed. Eng..

[8] Mezger U., Jendrewski C., Bartels M. (2013). Navigation in surgery. Langenbeck’s Arch. Surg..

[9] Ungi T., Gauvin G., Lasso A., Yeo C.T., Pezeshki P., Vaughan T., Carter K., Rudan J., Engel C.J., Fichtinger G. (2015). Navigated breast tumor excision using electromagnetically tracked ultrasound and surgical instruments. IEEE Trans. Biomed. Eng..

[10] Lo C.K., Li H.Y., Wong Y.C., Wai Y.L. (2018). Total knee replacement with iASSIST navigation system. J. Orthop. Trauma Rehabil..

[11] Frank T., Krieger A., Leonard S., Patel N.A., Tokuda J. (2017). ROS-IGTL-Bridge: An open network interface for image-guided therapy using the ROS environment. Int. J. Comput. Assist. Radiol. Surg..

[12] Herrell S.D., Galloway R.L., Su L.M. (2012). Image-guided robotic surgery: Update on research and potential applications in urologic surgery. Curr. Opin. Urol..

[13] Vladareanu V., Munteanu R.I., Mumtaz A., Smarandache F., Vladareanu L. (2015). The optimization of intelligent control interfaces using Versatile Intelligent Portable Robot Platform. Procedia Comput. Sci..

[14] Quigley M., Conley K., Gerkey B., Faust J., Foote T., Leibs J., Berger E., Wheeler R., Ng A.Y. ROS: An open-source Robot Operating System. Proceedings of the ICRA Workshop on Open Source Software.

[15] Chen Z., Deguet A., Vozar S., Munawar A., Fischer G., Kazanzides P. Interfacing the da Vinci Research Kit (dVRK) with the Robot Operating System (ROS). Proceedings of the 2015 IEEE International Conference on Robotics and Automation (ICRA).

[16] Tauscher S., Tokuda J., Schreiber G., Neff T., Hata N., Ortmaier T. (2015). OpenIGTLink interface for state control and visualisation of a robot for image-guided therapy systems. Int. J. Comput. Assist. Radiol. Surg..

[17] Ungi T., Lasso A., Fichtinger G. (2016). Open-source platforms for navigated image-guided interventions. Med. Image Anal..

[18] Pieper S., Halle M., Kikinis R. 3D Slicer. Proceedings of the 2004 2nd IEEE International Symposium on Biomedical Imaging: Nano to Macro (IEEE Cat No. 04EX821).

[19] Kikinis R., Pieper S. 3D Slicer as a tool for interactive brain tumor segmentation. Proceedings of the 2011 Annual International Conference of the IEEE Engineering in Medicine and Biology Society.

[20] Janssen N., Eppenga R., Peeters M.J.V., van Duijnhoven F., Oldenburg H., van der Hage J., Rutgers E., Sonke J.J., Kuhlmann K., Ruers T. (2018). Real-time wireless tumor tracking during breast conserving surgery. Int. J. Comput. Assist. Radiol. Surg..

[21] Schmidt K.F., Ziu M., Ole Schmidt N., Vaghasia P., Cargioli T.G., Doshi S., Albert M.S., Black P.M., Carroll R.S., Sun Y. (2004). Volume reconstruction techniques improve the correlation between histological and in vivo tumor volume measurements in mouse models of human gliomas. J. Neuro-Oncol..

[22] Mehrtash A., Pesteie M., Hetherington J., Behringer P.A., Kapur T., Wells W.M., Rohling R., Fedorov A., Abolmaesumi P. (2017). DeepInfer: Open-source deep learning deployment toolkit for image-guided therapy. Proceedings of the Medical Imaging 2017: Image-Guided Procedures, Robotic Interventions, and Modeling.

[23] Tokuda J., Fischer G.S., Papademetris X., Yaniv Z., Ibanez L., Cheng P., Liu H., Blevins J., Arata J., Golby A.J. (2009). OpenIGTLink: An open network protocol for image-guided therapy environment. Int. J. Med. Robot. Comput. Assist. Surg..

[24] Connolly L., Deguet A., Sunderland K., Lasso A., Ungi T., Rudan J.F., Taylor R.H., Mousavi P., Fichtinger G. An open-source platform for cooperative, semi-autonomous robotic surgery. Proceedings of the 2021 IEEE International Conference on Autonomous Systems (ICAS).

[25] Klotzbücher M., Soetens P., Bruyninckx H. Orocos RTT-Lua: An execution environment for building real-time robotic domain specific languages. Proceedings of the International Workshop on Dynamic languages for Robotic and Sensors.

[26] Stavrinos G. (2021). ROS2 For ROS1 Users. Robot Operating System (ROS).

[27] Gering D.T., Nabavi A., Kikinis R., Grimson W.E.L., Hata N., Everett P., Jolesz F., Wells W.M. (1999). An integrated visualization system for surgical planning and guidance using image fusion and interventional imaging. Proceedings of the International Conference on Medical Image Computing and Computer-Assisted Intervention.

[28] Foote T. tf: The transform library. Proceedings of the 2013 IEEE Conference on Technologies for Practical Robot Applications (TePRA).

[29] Kunze L., Roehm T., Beetz M. Towards semantic robot description languages. Proceedings of the 2011 IEEE International Conference on Robotics and Automation.

[30] Kazanzides P., Chen Z., Deguet A., Fischer G.S., Taylor R.H., DiMaio S.P. An open-source research kit for the da Vinci^®^ Surgical System. Proceedings of the IEEE International Conference on Robotics and Automation.

[31] Fontanelli G., Ficuciello F., Villani L., Siciliano B. Da Vinci research kit: PSM and MTM dynamic modelling. Proceedings of the IROS Workshop on Shared Platforms for Medical Robotics Research.

[32] Li M., Taylor R.H. Performance of surgical robots with automatically generated spatial virtual fixtures. Proceedings of the 2005 IEEE International Conference on Robotics and Automation.

[33] Welch H.G., Prorok P.C., O’Malley A.J., Kramer B.S. (2016). Breast-cancer tumor size, overdiagnosis, and mammography screening effectiveness. N. Engl. J. Med..

[34] D’Ettorre C., Mariani A., Stilli A., Valdastri P., Deguet A., Kazanzides P., Taylor R.H., Fischer G.S., DiMaio S.P., Menciassi A. (2021). Accelerating surgical robotics research: Reviewing 10 years of research with the dvrk. arXiv.

[35] Attig C., Rauh N., Franke T., Krems J.F. (2017). System latency guidelines then and now—Is zero latency really considered necessary?. Proceedings of the International Conference on Engineering Psychology and Cognitive Ergonomics.

